# Immunomodulatory-associated gene transcripts to multipotency of bovine amniotic fluid mesenchymal stem cells

**DOI:** 10.1590/1984-3143-AR2023-0155

**Published:** 2024-04-12

**Authors:** Jamila Cristina Baptistella, Carolina Gonzales da Silva, Sônia Nair Báo, Letícia Colin Panegossi, Tereza Cristina Cardoso, Roberto Gameiro de Carvalho, Carlos Frederico Martins

**Affiliations:** 1 Laboratório de Virologia e Cultura Celular, Faculdade de Medicina Veterinária, Universidade Estadual Paulista – UNESP, Araçatuba, SP, Brasil; 2 Faculdade de Medicina Veterinária, Centro Universitário Católico Salesiano – UniSalesiano, Araçatuba, SP, Brasil; 3 Instituto Federal de Educação, Ciência e Tecnologia da Bahia, Campus Xique-Xique, Xique-Xique, BA, Brasil; 4 Departamento de Biologia Celular, Instituto de Ciências Biológicas, Universidade de Brasília – UnB, Brasília, DF, Brasil; 5 Laboratório de Reprodução Animal, Empresa Brasileira de Pesquisa Agropecuária – Embrapa Cerrados, Planaltina, DF, Brasil

**Keywords:** bovine mesenchymal stromal cells, differentiation, immunomodulation, gene expression

## Abstract

The adnexa fetal tissues are sources of mesenchymal stromal cells (MSCs) due to their noninvasive harvest, with all biological material discarded most of the time. MSCs are a promise regarding to their plasticity, self-renewal, differentiation potentials, immunomodulatory and anti-inflammatory properties, which have made clinical stem cell therapy a reality. The present study aimed to characterize and evaluate the immunomodulation ability of bovine mesenchymal cells collected from bovine amniotic fluid (bAFMSCs) isolated and subjected to sixth consecutive culture passages *in vitro*. The multilineage properties of the bAFMSCs collections confirmed the ability to undergo adipogenic, chondrogenic and osteogenic differentiation. The mesenchymal gene transcription *CD106, CD73, CD29, CD90 and CD166* were detected in bAFMSCs, whereas *CD34* and *CD45* were not detected. Regarding cytokine mRNA expression, *IL2, IL6, INFα, INFβ, INFγ, TNFα* and *TNFβ* were downregulated, while *IL10* was highly regulated in all studied passages. The present study demonstrated the immunological properties and multipotency of *in vitro* bAFMSCs collections, and thus, they can be tested in cattle pathological treatments or multiplication by nuclear transfer cloning.

## Introduction

Bovine mesenchymal stem cells (bMSCs) have been derived from umbilical cord blood (bUCBMSCs), Wharton´s jelly (bWJMSCs), amniotic fluid (bAFMSCs), bone marrow (bBMMSCs) and fetal bovine liver sources (Raoufi et al., 2011; Corradetti et al., 2013). In fact, MSCs can interact and/or modulate the immune system *in vitro* among many biologicals models (Troyer and Weiss, 2008; Weiss et al., 2008; Prasanna et al., 2010; Miguel et al., 2012). Despite of many studies underlying the MSCs interference on immune system have been described in humans, canines, horses, goats, chickens, and pigs (Kang et al., 2008; Ankrum et al., 2014; Tessier et al., 2015; Saulnier et al., 2016), little is known about those in bovine species (Huaman et al., 2019).

Because of MSCs ability to modulate inflammatory responses, they are promising treatment agents in various inflammatory diseases (Somal et al., 2021). These cells promote tissue regeneration and healing by modulating the immune response, secreting growth factors, cytokines, and chemokines (Pieri et al., 2019), decreasing inflammation-associated cells and cytokines and increasing blood flow to promote normal healing instead of scarring (Peroni and Borjesson, 2011). Moreover, understanding the immunomodulatory and immunogenicity mechanisms of MSCs from domestic animals influences aspects related to human studies (Carrade and Borjesson, 2013).

In addition to their contribution to human medicine, animal MSCs and their benefits can be utilized for cattle pathological treatments, as example to repair post mastitis structural defects in dairy animals (Sharma and Jeong, 2013) and reproduction as an alternative method for cloned animals production (Silva et al., 2016). The aim of this study was to isolate MSCs from bovine amniotic fluid, characterize tri-lineage differentiation and molecular analysis of immunological properties by gene transcription.

## Methods

### Animals and cells collection

Chemicals and tissue culture plastic were purchased from Sigma‒Aldrich^®^ (St. Louis, MO, USA), Invitrogen (Invitrogen, California, USA), Applied Biosystems™ (Applied Biosystems^™^, CA, USA) and BD Falcon™ (BD Falcon, Bedford, USA) unless otherwise specified.

The Ethics Committee in Animal Use at the University of Brasília (protocol no. 151101/2013) approved all experimentation procedures.

Two gestations from cows (*Bos taurus indicus*) were established to obtain amniotic fluid cells. The amniocentesis, MSCs isolation and cell were performed according to the methods described previously (Silva et al., 2016). Ultrasound-guided transvaginal amniocentesis was performed to collect the amniotic fluid cells from two adult Guzerat cows, with pregnancies between 60 and 70 days. Briefly, a 7.5-MHz convex transducer was connected to a Honda ultrasound system (model HS1500V, Japan) and equipped with a 21-gauge needle 65 cm in length, localized in the tip transducer. When the needle reached the amniotic fluid, about 7 mL of fluid was aspirated with a 60-mL sterile syringe. A volume of amniotic fluid sufficient to maintain the pregnancy was preserved. The amniotic fluid was centrifuged at 135 xg for 10 min, and the supernatant was discarded. The sediment was resuspended in 3 mL of AmnioMAX-II Complete Medium (Gibco-BRL/Life Technologies, Rockville, MD, USA). The cells were cultured in special culture flasks in an incubator with 5% CO_2_, 90% humidity, and 38.5 °C until cellular confluence.

From initial culture, 1^st^, 3^rd^ and 6^th^ bAFMSCs passages were used for all experiments described above. In order to realize the cell differentiation bAFMSCs were culture in flasks (25cm^2^) coated with TissueCoat™ specific for adipose, cartilage and bone tissues (TissueLabs™, Manno, Switzerland) incubated in a humidified incubator at 38.5 °C with 5% CO_2_ atmosphere during 35 days. The cells were allowed to grow and were cultured by passaging after reaching >80% confluence at approximately 1×10^5^ cells/ml. The cell morphology and anchorage to culture plates were monitored and documented daily (Cunha et al., 2014). Undifferentiated cells were used for analysis as a negative control. After 35 days the respective cultures were fixed with 4% paraformaldehyde and processed to hematoxylin-eosin standard stain method.

### Cell viability and differentiation

Cell viability/proliferation was determined at the 1^st^, 3^rd^ and 6^th^ passages by MTS assay (Promega Corporation, Madison, WI, USA). Briefly, the isolated bAFMSCs were seeded in 96-well plates (1.5 × 10^4^ cells/well). Briefly, when bAFMSCs reached 80% confluence, MTS reagent (20 μL/well) was added, and the cells were incubated at 37 °C for 2 h. The absorbance was read at 490 nm using a microplate reader (Thermo Fisher Scientific, USA).

### Molecular analysis

Total RNA from bAFMSCs was extracted using TRIzol^®^ following the manufacturer’s recommendation. A total of 2 ng of each RNA sample was reverse-transcribed using the High Capacity RNA-to-cDNA Kit. RT‒qPCR to assess *CD106, CD73, CD29, CD90, CD166, CD44, CD45* and *CD34* transcripts. The expression of regulating genes was quantified using software on a StepOnePlus^®^ real-time instrument. The real-time PCR mixtures (50 µl) contained 1.2 µg of cDNA, 400 nM primers and 200 nM probes FAM-mGB (5` region) customized for *Bos taurus* bovine sequences. PCR was initiated by sequential amplification of 40 cycles at 95 °C (15 s) and 60 °C (60 s). The genes related to stem cell characterization, cell multipotency, and immunogenicity are described in Table 1. Major histocompatibility complex I (*MHCI*; Bt03279255_g1), *MHCII* (Bt03211217_m1), leptin (*LEP*; Bt03210417_m1), adipocyte fatty acid-binding protein (*FABP4*; Bt03213822_m1), peroxisome proliferator-activated receptor (*PPARD*; Bt03256949_m1), sex-determining region Y-box 9 (*SOX9*; (Bt04306555_m1), and collagen type 1 (*COL1A1*; (Bt03225349_g1)) were analyzed as multilineage differentiation transcripts in bAFMSCs.

**Table 1:** Specifications of (*Bos taurus*) cattle gene name searched by RT-qPCR.

**Gene symbol/ID**		**Description**
Positive markers of MSCs		
*ENG*	615.844	Endoglin (CD105)
*ITGB1*	281.876	Integrin, beta 1 (CD29)
*NT5E*	281.363	5`nucleosidase ecto (CD73)
*THY1*	614.712	Thy-1 cells surface antigen (CD90)
*CD34*	281.051	Hematopoietic progenitor cell antigen (CD34)
*PTPRC*	407.152	Protein tyrosine phosphatase, receptor type C (CD45)
*JSP.1*	407.173	Major histocompatibility complex class I (MHCI)
*DSB*	618.722	Major histocompatibility complex class II, antigen DS beta (MHC II)
Immune related genes		
*IFNAC*	281.236	Interferon alpha C (INF-alpha C)
*INFB1*	281.845	Interferon, beta 1, fibroblast
*IFNG*	281.237	Interferon, gamma
*IL2*	280.822	Interleukin 2
*IL6R*	507.359	Interleukin 6 receptor
*IL1F10*	615.702	Interleukin 1, family member 10
*TNF*	280.943	Tumor necrosis factor (TNF alpha)
*LTBR*	280.845	Lymphotoxin beta receptor
Positive markers of MSCs multipotency		
*LEP*	280.836	Leptin
*FABP4*	281.759	Fatty acid binding protein 4, adipocyte
*PPARD*	353.106	Perixome proliferator-activated receptor delta
*COL1A1*	282.187	Collagen type 1, alpha 1
*SOX9*	353.115	SRY (sex determining region Y)-box 10
*OMD*	280.885	Osteomodulin
*POST*	281.960	Osteoblast specific factor
*OSTF1*	281.961	Osteoclast stimulating factor 1
*GFAP*	281.189	Glial fibrillary acidic protein

Cytokine genes, including interferons *INFα* (Bt03215061_m1), *IFNβ* (Bt03278924_g1), and *INFγ* (Bt03212721_m1), interleukins *IL2* (Bt03217367_g1), *IL6* (Bt03211904_m1), and *IL10* (Bt03212726_m1), and tumor necrosis factors *TNFα* (Bt03259155_g1) and *TNFβ* (Bt03258737_g1), as well glial fibrillary acid protein (*GFAP*; Bt03251656_g1), were also analyzed.

### Statistical analysis

Quantification of gene expression was performed by the 2 ^–^*^ΔΔ^*^Ct^ method using the bovine histone 2a gene *H2A* (Bt03252057_g1) as a housekeep gene to normalize the results. The data are expressed as a relative gene expression, which indicates the fold change in gene expression to demonstrate whether the gene was upregulated or downregulated in bAFMSCs. Statistical analysis was performed using GraphPad Prism 9.3.1 for Windows (GraphPad Software, La Jolla, CA, USA). Four replicates were performed for each experiment, and the results are reported as the mean ± s.d. One-way ANOVA for multiple comparisons. P < 0.05 was considered significant.

## Results

### Cell characterization

**Figure 1:**
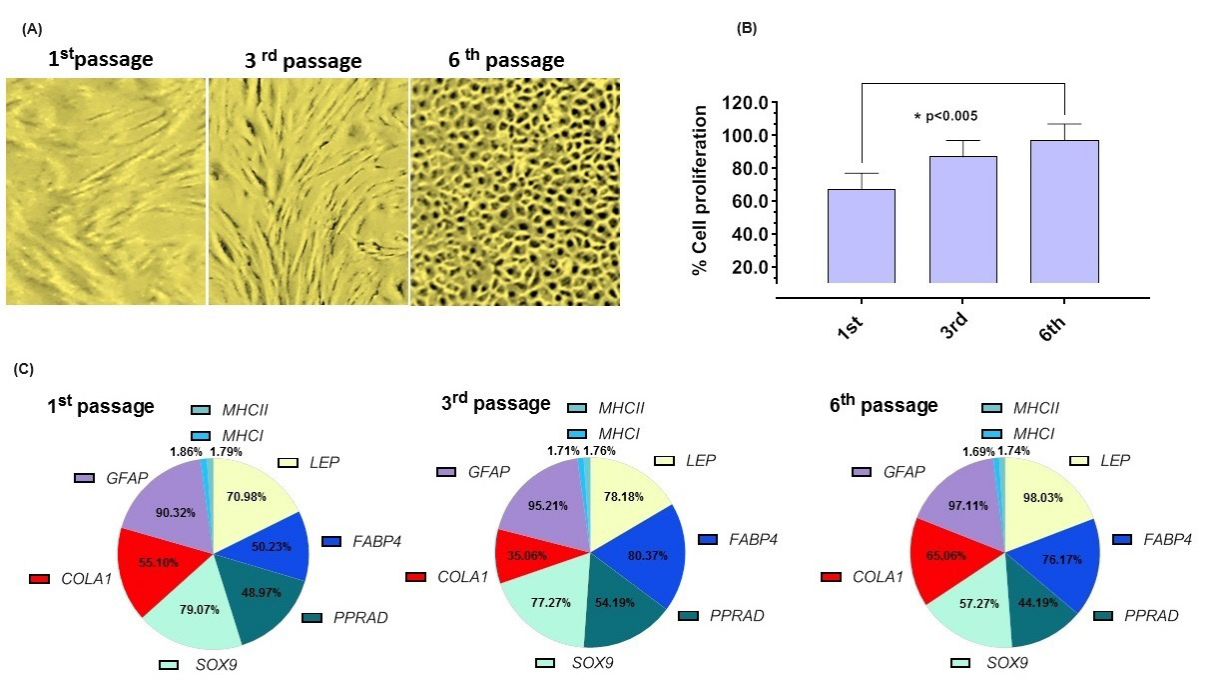
(A) Representative photomicrographs isolated from bAFMSCs at 1^st^, 3^rd^ and 6^th^ taken under phase contrast microscopy (40-μm of magnification); (B) Graph bars sowing bAFMSC proliferation rates increasing under cell passages; (C) Percentage of bAFMSCs positive for differentially expressed genes at the 1^st^, 3^rd^ and 6^th^ consecutive *in vitro* passages.

**Figure 2:**
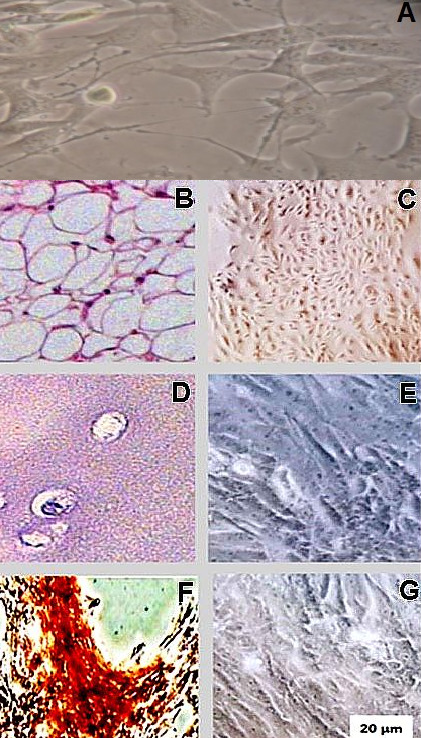
Multipotency assessment of the bAFMSCs revealed their adipogenic (B and C), chondrogenic (D and E) and osteogenic (F and G) differentiation. The **figure sho**ws differentiated cells stained by hematoxylin and eosin standard procedure and their respective 3D images. The **Figure 2A** is showing bAFMSCs before of differentiation (Control).

Bovine mesenchymal amniotic fluid cells (bAFMSCs) exhibited a fibroblast-like morphology and 80-90% cellular confluence after the 1^st^, 3^rd^ and 6^th^ consecutive passages in culture (Figure 1A). The bAFMSCs proliferation rate was calculated, and at the 6^th^ consecutive passage, a high percentage of viable cells was detected (Figure 1B). The multipotency assessment of the bAFMSCs revealed their adipogenic, chondrogenic and osteogenic at 6^th^ passage after 35 days (Figure 2). RT‒qPCR revealed the transcription of the *LEP, FABP4, PPRAD, SOX9* and *COLA1* genes at high levels during the cell passaging (Figure 1C). In contrast, *MHCI* and *MHCII* were not transcribed. The ectodermal potency of differentiation was demonstrated by *GFAP* transcription (Figure 1C).

### Transcriptional analysis

**Figure 3:**
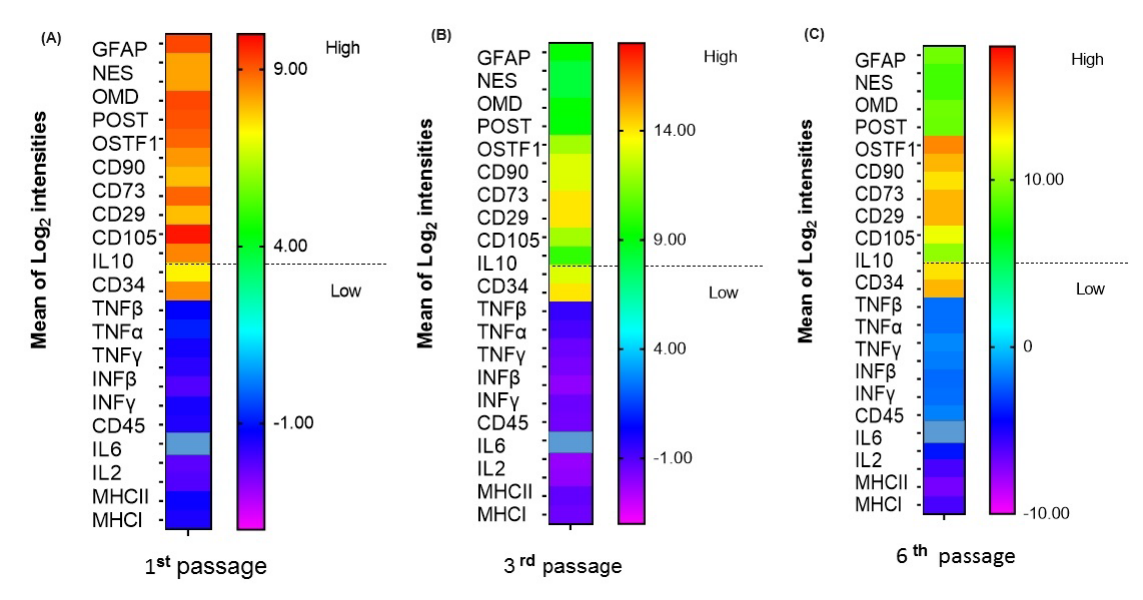
Heatmap of differentially expressed genes shows the average signal by bAFMSCs at the 1^st^, 3^rd^ and 6^th^ consecutive *in vitro* passages (A, B and C). The red color indicates increased expression, and the yellow to blue color indicates decreased expression compared to the control (n = 3/repetition; p value*<*0.01; linear fold change > 2); transcription analysis of bAFMSC immune-related genes. Expression of cytokines *IL2, IL6, INFα, INFβ, IFNγ, TNFα* and *TNFβ* was downregulated, while *IL10* was highly expressed in all tested bAFMSCs.

Analysis of genes typically used for MSC characterization revealed results consistent with the flow cytometric analysis. High transcription levels of the MSC markers *THY1* (CD90), *NT5E* (CD73), *ITGB1* (CD29), *and ENG* (CD105) and low levels of *CD34* and *PTPRC* (CD45) were found among the bAFMSCs at all passages (Table 1; Figures 3A, B and C). When stimulated to differentiate toward adipogenic, chondrogenic, osteogenic and neurogenic lineages, bAFMSCs showed substantial transcriptional expression of *LEP, FABP4, PPARD, COL1A1, SOX9, and GFAP* for all passages of bAFMSCs (Figure 3). The bAFMSCs’ potential to undergo chondrogenesis was shown by a high level of *COL1A1* gene expression (Figure 3). From the factors measured in this study, *IL2, IL6, INFα, INFβ, INFγ, TNFa* and *TNFβ*, considered proinflammatory cytokines, were downregulated in all bAFMSCs tested at all passages analyzed (Figure 3; p<0.005). However, *IL10* was noticeably upregulated, as shown by RT‒qPCR (Figure 3). The lack of *JSP.1* and *DSB* (MHCI and II) expression could be observed in all passages (Figure 3).

## Discussion

Tissues derived from extra gestational sources have been suggested as ideal of mesenchymal cells due to their noninvasive harvest, with all biological material being discarded most of the time (Troyer and Weiss, 2008). Since little information is available on the immunomodulatory and immunogenic roles of MSCs derived from bovine fetal adnexa, the aim of this study was to characterize immunological properties of MSCs obtained from amniotic fluid in bovine species.

Cultured bovine mesenchymal amniotic fluid cells at the 1^st^, 3^rd^ and 6^th^ consecutive passages exhibited a fibroblast-like morphology and grew to 80-90% confluency. These results are in agreement with those described previously for bovine MSCs (Raoufi et al., 2011; Corradetti et al., 2013). Herein, results indicated that bAFMSCs expressed *CD106, CD73, CD29, CD90,* and *CD166.* However, the expression of *CD34* and *CD45* were negative in the tested bAFMSCs. Overall, the gene expression profile in the bAFMSCs shown in this study is similar to what was described in other studies (Prasanna et al., 2010).

The *in vitro* differentiation results support the findings already reported in bAFMSCs [2]. The bAFMSCs showed high plasticity and were able to differentiate into multiple germ layers of mesoderm and ectoderm. The results are in agreement with all studies regarding bovine MSCs (Tessier et al., 2015; Saulnier et al., 2016). Besides, MSCs stimulated to differentiation toward the chondrogenic lineage expressed a high level of *COLA1* transcription at the 3^rd^ passage (Corradetti et al., 2013).

Besides, there are contradictory information regarding to human MSCs immunogenicity and a lack of studies about bovine MSCs. However, porcine umbilical cord-derived stem cells not induce a considerable immune response *in vivo,* but when stimulated with interferon gamma (INFγ) or injection into an inflamed region resulted in immunogenicity (Poncelet et al., 2007; Prasanna et al., 2010).

The immunomodulatory properties of bAFMSCs include proinflammatory cytokines production, such as INFƴ and tumor necrosis factor alpha (TNFα), in the inflammatory microenvironment (Kode et al., 2009; Di Trapani et al., 2013). The most important immunosuppressive factors are indoleamine 2,3-dioxygenase (IDO), prostaglandin E2 (PGE2), nitric oxide (NO), transforming growth factor beta (TGFβ), HGF, IL10, IL1Ra and growth-related oncogene (GRO) (Kode et al., 2009; Di Trapani et al., 2013). In this study, the upregulation of *IL10* in bovine AFMSCs was proven even without previous exposure of the cells to INFƴ. These results corroborate those found in bovine fetal cells derived from bone marrow (BMMSCs) and from adipose tissue (ATMSCs), where *IL10* expression did not change significantly in the presence or absence of IFNƴ in the culture of these cell types (Huaman et al., 2019).

Therefore, all immune soluble mediators measured in this study, *IL2, IL6, INFα, INFβ, INFγ, TNFα* and *TNFβ*, considered proinflammatory cytokines, were genetically downregulated in bAFMSCs. However, when *IL10* is expressed, it downregulates the *MHCI* gene, as revealed in the bAFMSC culture. These results are in accordance with what has been described previously in human MSCs (Mukonoweshuro et al., 2014; Huaman et al., 2019). In contrast, MSCs from the amniotic sac, amniotic fluid, Wharton’s jelly and goat umbilical cord blood did not show significant changes in *IL6* mRNA expression after stimulation with *IFNƴ* and *TNFα* (Somal et al., 2021).

Moreover, previous studies revealed that MSCs have immunosuppressive properties; however, they are not immunoprivileged (Huaman et al., 2019).The lack of *MHCII* and low *MHC* expression observed in this study in bAFMSCs are thought to be in part responsible for their immunoprivileged status.

## Conclusion

The present study demonstrated the immunomodulatory potential and properties of multipotency *in vitro* of bovine AFMSCs. It is also necessary to expand our knowledge to investigate how bovine AFMSCs interact with other cells of the immune system, as well as correlate these findings with *in vivo* experiments. These findings demonstrated the complexity of bAFMSCs immunological properties *in vitro* and the difficulty of distinguishing among mesenchymal marker expression in different species. Furthermore, AFMSCs constitute a new cellular type with potential to be studied for cattle pathological treatments, as well as for multiplication through cloning by nuclear transfer.
